# A Nitric Oxide-Responsive Quorum Sensing Circuit in *Vibrio harveyi* Regulates Flagella Production and Biofilm Formation

**DOI:** 10.3390/ijms140816473

**Published:** 2013-08-08

**Authors:** Bernadette M. Henares, Yueming Xu, Elizabeth M. Boon

**Affiliations:** Department of Chemistry and Institute of Chemical Biology & Drug Discovery, Stony Brook University, Stony Brook, NY 11794-3400, USA; E-Mails: bhenares@gmail.com (B.M.H.); beiyouyu@gmail.com (Y.X.)

**Keywords:** nitric oxide, biofilm, quorum sensing, flagella, bacterial motility, H-NOX

## Abstract

Cell signaling plays an important role in the survival of bacterial colonies. They use small molecules to coordinate gene expression in a cell density dependent manner. This process, known as quorum sensing, helps bacteria regulate diverse functions such as bioluminescence, biofilm formation and virulence. In *Vibrio harveyi*, a bioluminescent marine bacterium, four parallel quorum-sensing systems have been identified to regulate light production. We have previously reported that nitric oxide (NO), through the H-NOX/HqsK quorum sensing pathway contributes to light production in *V. harveyi* through the LuxU/LuxO/LuxR quorum sensing pathway. In this study, we show that nitric oxide (NO) also regulates flagellar production and enhances biofilm formation. Our data suggest that *V. harveyi* is capable of switching between lifestyles to be able to adapt to changes in the environment.

## 1. Introduction

Most bacteria are capable of switching between a free-swimming, solitary, planktonic growth mode and sessile, biofilm living [1]. Biofilms are communities of surface-associated bacteria that form a complex, yet ordered, three-dimensional structure encapsulated in a self-secreted extracellular polysaccharide matrix (EPS). Members of a biofilm community have advantages such as access to nutrients, resistance to antimicrobial agents and protection against predators [2–4]. Biofilm formation is associated with quorum sensing (QS). Quorum sensing refers to the process by which bacteria coordinate social behaviors by secreting, detecting and responding to small molecules called autoinducers (AI) [5]. The sequential steps involved in biofilm formation require regulation of many genes across the bacterial community [1]; thus, efficient communication is required. To achieve this remarkable feat, bacteria send out signals as soon as they enter the confines of the biofilm, since proximity to each other makes it more feasible for bacteria to communicate with one another and engage in the maintenance of the well being of the community [5].

*Vibrio harveyi* is widely studied as a model bacterium for understanding quorum sensing. *V. harveyi* are well understood to regulate the gene expression of community behaviors such as bioluminescence and virulence gene production using QS circuits [6–8]. Furthermore, *V. harveyi*, are known to exist as free-swimming single cells, attached to abiotic surfaces as a biofilm, and in association with a host as a pathogen of marine animals [9]. Although it has been shown that *V. harveyi* can switch between these lifestyles, limited information is available about biofilm formation in *V. harveyi*, and there are even fewer studies that correlate QS and biofilm formation.

*V. harveyi* have (at least) four parallel quorum sensing sensor-kinases, each of which responds to detection of its cognate AI by a change in kinase activity [10]. Each of these kinases contributes to regulating the phosphorylation state of a common phosphorelay protein called LuxU [10,11]. LuxU transfers phosphate to and from LuxO [12], a transcriptional regulator that controls the expression of LuxR, and ultimately, the quorum sensing response. We previously reported that one of the four known QS pathways responds to nitric oxide (NO) through the H-NOX/HqsK (heme-nitric oxide/oxygen binding domain; H-NOX-associated quorum sensing kinase) sensor/kinase pair [10]. We have shown that NO acts analogously with the other AIs and positively regulates light production.

Several studies have shown that LuxR and its homologues indirectly regulate biofilm formation through QS. Genetic studies done on *V. parahaemolyticus*, *V. vulnificus* and *V. fischeri* reveal that QS positively regulates biofilm formation [13,14] through OpaR, SmcR, and LitR [15] (homologs of *V. harveyi* LuxR), respectively. An opposite regulation is observed in *V. cholerae* where vibrio polysaccharide (*VPS*, equivalent of *EPS*) gene expression is more abundant in the absence of HapR (LuxR homolog in *V. cholerae*), indicating a negative regulation of biofilm formation [16–18]. The ecological importance of this regulation in *V. cholerae* is still being investigated, but it has been suggested that being able to detach from the community is important in the transmission, colonization, and persistence of the next generation. Moreover, in a genetic study, *luxR*, expressed as a function of AI concentration, is shown to negatively regulate the expression of flagellar operons in *V. harveyi* [19,20]. A single polar flagellum provides bacteria an effective means of motility. In the initial stages of biofilm formation in *Vibrio*, attachment to a surface has been shown to involve the loss of flagellar genes [21]. Thus a loss of the flagellum is predicted to upregulate biofilm formation.

Interestingly, NO is well known to be involved in biofilm formation. Indeed, in many bacterial species such as *Shewanella woodyi* [22], *Shewanella oneidensis* [23], and *Legionella pneumophila* [24], NO is detected by H-NOX, which goes onto regulate biofilm formation through a pathway involving cyclic-di-GMP metabolism. Cyclic-di-GMP is a second messenger widely used by bacteria to regulate biofilm formation and EPS production [25,26]. In these systems, NO is sensed by H-NOX and ultimately regulates the activities of a diguanylate cyclase and/or phosphodiesterase, either directly [22,24], or indirectly through a histidine kinase [23], to control the intracellular concentration of cyclic-di-GMP.

In *Pseudomonas aeruginosa*, NO causes QS-mediated biofilm dispersal [27]. Thus we hypothesized that NO/H-NOX might contribute to regulation of biofilm formation through QS in *V. harveyi.* Here, using genetic, biofilm, and proteomic data, we demonstrate that NO/H-NOX regulates biofilm and flagellar formation in *V. harveyi.*

## 2. Results and Discussion

We have shown in our previous work that NO enhances light production at the initial stage of bioluminescence through LuxR and the QS pathway [10]. However, the *lux* operon is not the only set of genes regulated by QS [28]. Thus we hypothesize that other than light production, NO may also regulate biofilm formation by entering the QS pathway through the H-NOX/HqsK circuit. Our hypothesis is based on several literature observations. First, NO is well understood to be involved in biofilm formation in a wide range of bacteria [22–24], and in *P. aeruginosa*, NO has been reported to affect biofilm formation through QS processes [27,29]. Furthermore, several lines of evidence in several strains of *Vibrio* have demonstrated that LuxR and QS indirectly regulate biofilm formation and expression of the flagella operon [21]. Thus, we expect QS to upregulate biofilm formation in the presence of NO.

**Figure 1:**
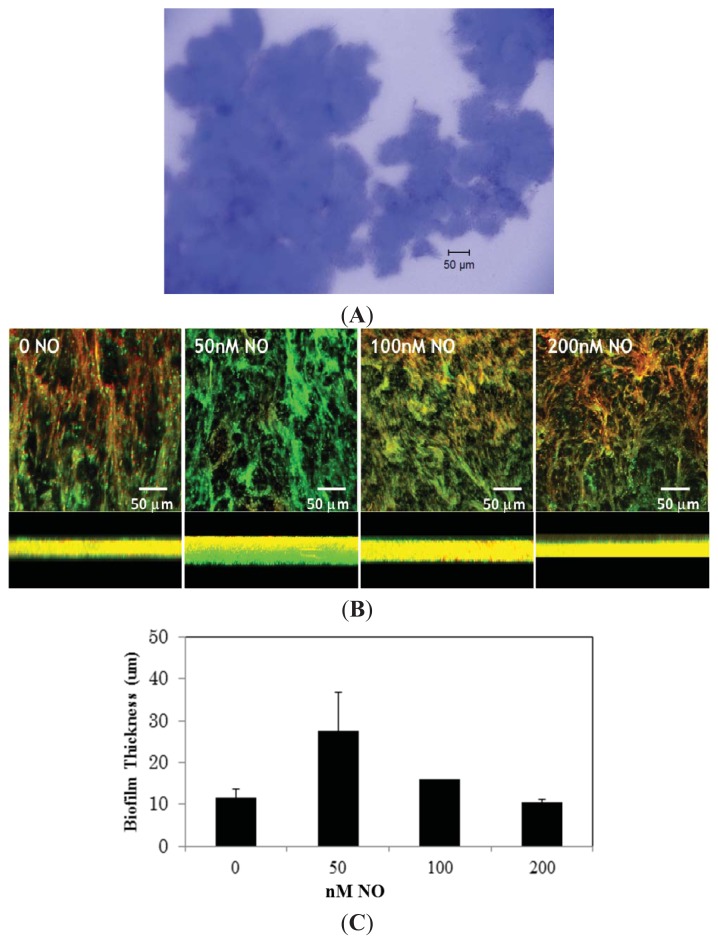
Effect of nitric oxide (NO) on biofilm thickness and cell viability, quantified by confocal laser scanning microscope (CLSM). (**A**) Phase-contrast image of a biofilm at 10× magnification stained with calcofluor to show extracellular polysaccharide matrix (EPS) production. This biofilm was grown without the addition of NO; (**B**) Confocal images of biofilm at 40× magnification, grown in the presence of the indicated amount of NO. Cells were stained with SYTO 9 (green; stains all cells) and propidium iodide (red; stains dead cells only). Top pictures are the *x*–*y* view, bottom pictures show the *y*-dimension as viewed on the side; (**C**) Summary of biofilm thickness as a function of NO concentration.

**Figure 2:**
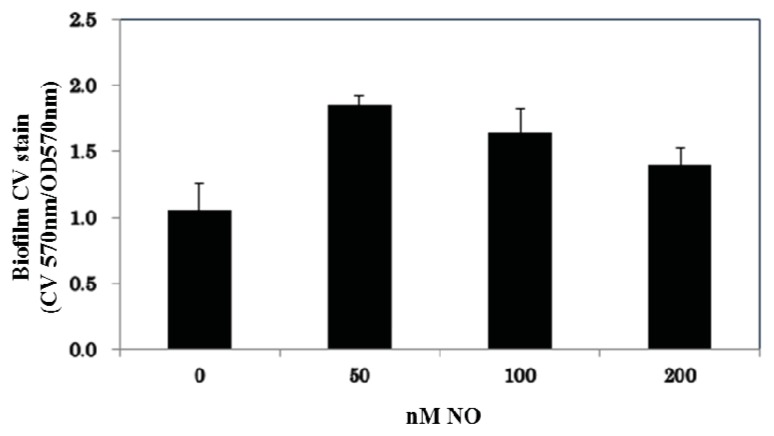
Effect of NO on biofilm formation in *V. harveyi* WT strain quantified using the crystal violet staining method. Normalized crystal violet (CV) is reported as the CV absorbance at 570 nm divided by OD of all cells, planktonic and biofilm, at 570 nm. Error bars represent one standard deviation from the mean of triplicate experiments.

To test this hypothesis, we investigated the effect of NO on biofilm formation by *V. harveyi* wild-type strain BB120. Using concentrations of NO that have no effect on a planktonic growth curve (Supplementary Figure 1), biofilm architecture, biofilm thickness, and cell viability were determined using confocal laser scanning microscope (CLSM). The cells were allowed to grow on microscope slides for 12 h at 30 °C. Biofilms that formed at the air-liquid interface were visualized by staining with SYTO 9 (green; stains live cells) and propidium iodide (red; stains dead cells only) for observation under confocal microscope while EPS production was viewed using calcoflour white under the phase-contrast method. Under the conditions in which the biofilms were obtained, most cells were viable and *V. harveyi* were able to form EPS and thick, biofilms under aerobic conditions (Figure 1A). Individual cells are difficult to see due to other substances present in the EPS matrix (DNA, protein). Cells exposed to 50 nM NO showed a remarkably thick biofilm in comparison to the culture grown without added NO (Figure 1B,C). On the other hand, biofilm formation goes back to the without NO thickness at NO concentrations exceeding 100 nM. This observation was corroborated when we quantified biofilm formation of *V. harveyi* grown in 96-well plates using the crystal violet staining method [19]. As illustrated in Figure 2, a similar trend was observed: at 50 nM NO we observed more biofilm formation than in the absence of NO, while at higher NO concentrations, biofilm levels decreased.

It is not clear why there is a concentration-dependent switch in biofilm regulation in response to NO, although this NO phenotype has been previously observed in *Nitrosomonas europea*, where biofilm formation is induced at 30 ppm while a NO concentration below 5 ppm promotes dispersal [29]. It is possible that at higher NO concentrations, NO is detected by a less sensitive NO sensor that regulates an alternate biofilm response. It is also possible that NO, through H-NOX, induces dispersal of biofilm through a different (not QS) pathway. For example, like many histidine kinases, HqsK could transfer phosphate to more than one response regulator, thus feeding into several pathways.

**Figure 3:**
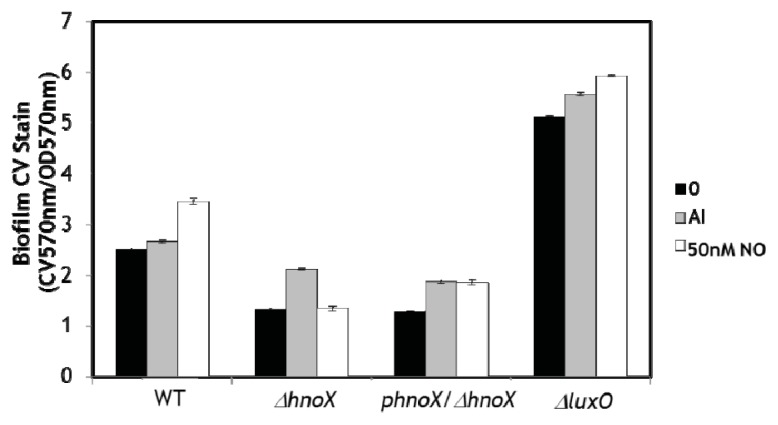
Effect of NO on biofilm formation in *V. harveyi* WT, Δ*hnoX*, *phnoX*/Δ*hnoX*, and Δ*luxO* strains. Normalized CV is reported as the CV absorbance at 570 nm divided by OD of all cells, planktonic and biofilm, at 570 nm. The black bars indicate no additives, the grey bars indicate the addition of cell-free medium from an overnight culture, which is rich in AIs, and the white bars indicate the addition of 50 nM NO. Error bars represent one standard deviation from the mean of triplicate experiments.

These data demonstrate that NO enhances biofilm formation in *V. harveyi* at low concentration, consistent with our hypothesis that biofilm is positively regulated by NO/H-NOX, possibly through QS. Next we investigated the effect of NO on biofilm formation of WT and several QS mutants. As shown in Figure 3, biofilm formation is enhanced in WT cultures grown in the presence of 50 nM NO, which is consistent with our CLSM and CV assays discussed above. As expected, deletion of the *hnoX* gene results in decreased biofilm and does not elicit the same biofilm enhancement in the presence of NO, indicating that H-NOX positively regulates biofilm formation. Furthermore, complementation of the Δ*hnoX* mutant strain with H-NOX expressed on a plasmid (the Δ*hnoX*/*phnoX* strain) restores the NO-induced increase in biofilm phenotype. These data confirm that H-NOX is the NO sensor responsible for the increase in biofilm in the presence of NO. Interestingly, the addition of cell-free medium from an overnight culture, which contains a high concentration of all the autoinducers that trigger QS pathways in *V. harveyi* (+AI), does not result in as large an increase in biofilm as NO, suggesting that NO/H-NOX is the primary QS circuit affecting biofilm formation.

The mutant strain Δ*luxO*, which is used as positive control for QS, showed a much higher biofilm than WT, verifying that QS and LuxR positively regulate biofilm formation. This strain contains a Δ*luxO* mutation which renders the production of LuxR independent of AI concentration, thus this strain is constitutively bright and locked at the high cell density state. The addition of NO and excess AI do appear to further increase biofilm formation, although these small increases in CV staining could be due to the error associated with dividing a relatively large number (CV absorbance) by a relatively small number (cell density; although there are more biofilm cells in the Δ*luxO* strain, the total cell density is comparable for all samples in this experiment). It is also possible that there is an alternative NO-mediated pathway that affects biofilm formation, as discussed above. However, because there is no effect on NO in the Δ*hnoX* mutant, we do not believe this is the most likely explanation. Taken together, these results are supportive of NO-mediated regulation of biofilm formation through QS via the H-NOX/HqsK system. In our previous studies we demonstrated that H-NOX responds to NO by regulating the flow of phosphate into the QS circuitry through the H-NOX-associated quorum sensing kinase (HqsK).

To support our biofilm analysis, we performed an iTRAQ proteomics analysis on *V. harveyi* exposed to varying concentrations of NO. In our analysis, we identified a total of 529 proteins from ~4800 predicted proteins in the *V. harveyi* genome. Of the 529 proteins, 493 were identified by two or more significant peptides. Protein mixtures obtained after growth in the presence of 0, 50, 100, or 200 nM NO were labeled with isobaric tags that produce signature ions at *m*/*z* 114, 115, 116, and 117, respectively. The effect of NO on the proteome was determined based on the ratio of an isobaric tag ion peak at a given NO concentration over that same tag peak area in the absence of NO (Supplementary Table 1). We selected proteins whose expression ratios fell outside of 1.000 ± 0.2 as being significantly affected by NO.

**Figure 4:**
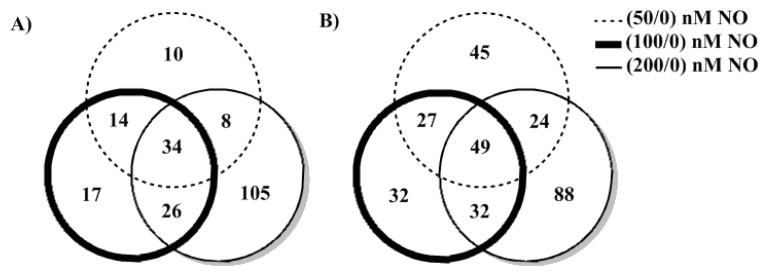
Summary of *V. harveyi* iTRAQ analysis. Proteins that were down-regulated (**A**) or upregulated (**B**) by at least 20% were selected and compared under different NO concentrations. Numbers in the common areas indicate proteins that show the same trend as a function of NO concentration. Remaining numbers represent proteins that are up- or down-regulated only under one condition.

A number of proteins displayed a NO concentration-dependent trend. In Figure 4, these peptides are indicated in the overlapping areas of the Venn diagram. Among the downregulated proteins (Figure 4A), 65.9% of 116/114 (100 nM/0 nM NO) also showed up in 117/114 (200 nM/0 nM NO), while 52.7% overlapped with 115/114 (50 nM/0 nM NO). 57.8% and 54.3% of upreglated proteins (Figure 4B) overlapped with 115/114 and 117/114, respectively. Some protein levels were strongly decreased at 50 nM NO, but were restored as NO concentration was increased. Therefore, the pattern of proteome at 50 nM is slightly different from 100 and 200 nM. A higher similarity between 100 nM/0 nM NO and 200 nM/0 nM NO might be an indication of a NO concentration dependent switch of bacterial protein expression between 50 and 100 nM NO. Notably, this is the same NO-dependent pattern we observed for biofilm formation, indicating there is possibly a global switch that takes place in protein expression as NO is increased from 50 to 100 nM.

Here we highlight and further analyze several proteins that are known to be involved in biofilm formation (Table 1). All five *V. harveyi* flagellin proteins display the same NO-concentration dependent trend. They show a significant decrease at 50 nM NO and are restored to the same levels as without NO as the NO concentration is increased. A functional flagella has been proven to be critical in initial attachment [30–32] of Gram-negative bacteria and early exopolysaccharide synthesis. Furthermore, CheY protein expression is unchanged at 50 nM NO, but is repressed upon exposure to higher NO concentration. CheY can bind to FilM at the base of flagellar motor and modify flagellar behavior [33]. It is reported in *E. coli* that an overexpression of CheY can enable clockwise rotation [34–36] and reduce bacteria motility. Although the experiments were carried out with agitation and no biofilm was observed, when taken together, these results imply an upregulation in biofilm due to a decrease in motility and enhanced surface attachment at low NO concentration, followed by a return to normal motility as NO continues to increase. Interestingly, this is exactly the same trend that we observed in our biofilm analysis: an increase in biofilm at low NO concentration followed by a decrease as NO concentration is increased. In the presence of NO, flagellin and CheY might synergistically contribute to bacterial initial attachment. A microarray study in *Vibrio fischeri* has shown that several flagellins and flagellar basal-body proteins are negatively regulated by quorum sensing system [37]. Based on our results, we suggest that in *V. harveyi*, NO regulates flagella production through QS.

## 3. Experimental Section

### 3.1. Bacterial Strains and Growth Conditions

Strains used in this study are listed in Table 2. *V. harveyi* strains wild-type (WT), Δ*luxO*, and Δ*luxNS* were purchased from the American Type Culture Collection (ATCC). *V. harveyi* mutants Δ*hnoX* and *phnoX/*Δ*hnoX* are lab strains constructed as previously described [10]. Cell cultures were maintained in marine media (MM; 28 g/L; BD Difco, Sparks, NV, USA) and grown at 30 °C with agitation at 250 rpm.

### 3.2. Biofilm Imaging by Confocal Microscopy

Microscopy images were recorded on a Zeiss LSM 510 Metanlo Two-Photon Laser Scanning Confocal Microscope System (Carl Zeiss, Jena, Germany). An overnight culture of *V. harveyi* in marine media was diluted (1:1000) into fresh medium in a sterile 50 mL conical centrifuge tube containing a glass microscope slide. The medium also contained various concentrations of DPTA NONOate that had been predecayed for 3 h at 30 °C, resulting in 0, 50, 100, and 200 nM NO in solution, as measured by a Sievers nitric oxide analyzer (NOA 280i, GE analytical instruments, Boulder, CO, USA). Biofilms were grown under static conditions at 30 °C with slow agitation at 50 rpm for 12 h. Following the growth period, the slide was thoroughly rinsed with distilled water and the adhered biofilm cells were stained for imaging. Samples were stained with the 1% calcoflour white for 15 min to stain and image EPS. Cells for confocal microscopy were stained with LIVE/DEAD BacLight kit (Invitrogen, Carlsbad, California) according to the manufacturer’s protocol, for 15 min. The biofilm formed at the air–liquid interface was then imaged and analyzed. The air–liquid interface was ~3 mm wide (in the *X* dimension, along the longest side of the microscope slide), as determined from crystal violet staining of identically obtained biofilms on microscope slides. The biofilm thickness (*X*−*Z* dimension, *i.e.*, the height of the biofilm measured from the surface of the microscope slide to the top of the biofilm) was measured at three different locations in each experiment and averaged to determine the mean biofilm thickness. The locations were chosen randomly, but generally one spot near the middle of the slide and one from each edge of the slide (in the *Y* dimension) were chosen. Multiple locations were measured because bacterial biofilms are often not of uniform thickness. These measurements may not account for all the variation in biofilm thickness, but they provide an estimate of biofilm thickness for comparison between different NO concentrations. Confocal images for each of three completely independently grown biofilms exposed to each NO concentration were separately grown and analyzed. The mean thickness from each trial was determined from measurements at multiple locations. The mean thickness from three independent trials ± one standard deviation is reported.

### 3.3. Crystal Violet Staining for Biofilm Quantification

Steady-state biofilm formation, at the air-liquid interface, in a shaking culture was examined in 96-well polyvinyl chloride (PVC) plates as previously described [22], with a few modifications. A 100 μL subculture (1:100 dilution of an overnight culture of *V. harveyi*) in marine media was incubated at 30 °C for 12 h with slow agitation (50 rpm). Some cultures included the addition of 50 nM NO (from NONOate) or cell-free medium from an overnight culture (contains a high concentration of AIs). The planktonic cells and media were then removed, and the remaining biofilm was rigorously washed with water followed by staining with 150 μL of 0.1% crystal violet (CV) in water for 15 min. Next the CV solution was removed, and the wells were rinsed three times with distilled water and allowed to thoroughly dry. Then 100 μL of DMSO was added to each well to solubilize the CV adsorbed by the biofilm cells. The DMSO/CV solution was removed from the PVC plate and added to a polystyrene 96-well plate, and the optical density at 570 nm was measured with a Perkin-Elmer Victor X5 multilabel reader (Perkin Elmer, Waltham, MA, USA). The data are reported as the CV absorbance at 570 nm divided by the optical density of the planktonic and biofilm cells at 600 nm. Each biofilm condition was run a minimum of 10 times in one experiment, and the entire experiment was independently performed a minimum of three times. The mean measurement ± one standard deviation from 3 independent experiments is reported.

### 3.4. iTRAQ™ Analysis of *V. harveyi*

An overnight *V. harveyi* culture was inoculated (1:100 dilution) into autoinducer bioassay media (AB; 0.2% vitamin-free casamino acids, 0.3 M NaCl, 0.05 M MgSO_4_, 10 mM potassium phosphate pH 7.0, 1 mM l-arginine, 1% (*v*/*v*) glycerol) [38] supplemented with DPTA NONOate (Cayman Chemical, San Diego, CA, USA) that had been predecayed for 3 h at 30 °C, resulting in 0, 50, 100, and 200 nM NO in solution, as measured by a Sievers nitric oxide analyzer (NOA 280i, GE analytical instruments, Boulder, CO, USA). After 10 h, the culture was harvested by centrifugation at 5000 rpm at 4 °C (Beckman Coulter, Brea, CA, USA) and re-suspended in 300 μL Milli-Q water followed by repeated freeze–thaw cycles in liquid nitrogen. Cell lysates were treated by acetone precipitation and protein samples were re-suspended in dissolution buffer using the iTRAQ™ kit (Applied Biosystems, Foster City, CA, USA). Protein mixtures were reduced and alkylated according to manufacturer’s protocol. The total protein concentrations were then quantified using bicinchoninic acid (BCA) assay kit (Thermo Scientific, Rockford, IL, USA). For each sample, 100 μg of total protein was digested with trypsin (Roche Diagnostics, Mannheim, Germany) at 37 °C overnight and labeled with iTRAQ™ reagents. Then samples with different labels (corresponding to samples grown in different NO concentrations) were mixed together. Protein samples were analyzed on a Thermo Fisher Scientific LTQ Orbitrap XL ETD by the proteomics core facility center at Stony Brook University.

## 4. Conclusions

In the natural environment, bacteria are often part of a multicultural community. They spend most of their time in social communes where they are covered with an EPS matrix that confers protection against environmental stress. Biofilm regulation is not well understood, although cyclic-di-GMP signaling and QS are known to play a role [39,40]. A global regulatory mechanism is needed to achieve biofilm formation because it involves the concomitant expression and repression of tens or even hundreds of unlinked genes in a cell-density dependent manner [41]. It appears that QS reciprocally influences cyclic-di-GMP signaling pathways, together providing an integrated network for assimilating numerous external stimuli into a community-wide response [42,43].

Our data suggest that, at low concentration, NO acts as a stimulus to promote *V. harveyi* biofilm formation. In several bacteria, NO/H-NOX regulates cyclic-di-GMP synthesis and/or hydrolysis to contribute to EPS production and biofilm regulation [22–24]. Here, we report QS-mediated biofilm formation through regulation of flagellar proteins via the H-NOX/HqsK pathway. It is interesting that all NO/H-NOX pathways characterized to date are involved in biofilm regulation, although the details of the signaling pathway appear to vary from organism to organism. These results serve as a starting point to study in detail the mechanism involved in formation of biofilms through QS. Biofilms involve community-wide changes in gene expression, and thus QS plays a critical role that is still being uncovered.

## Supplementary Information



## Figures and Tables

**Table 1:** Selected biofilm-associated proteins as a function of NO.

Gene ID	Protein ID	50 nM NO fold change a	100 nM NO fold change a	200 nM NO fold change a	Annotation
Vibhar_01300	A7MT73	0.364	0.625	0.813	Flagellin
Vibhar_01301	A7MT74	0.421	0.637	1.012	Flagellin
Vibhar_03171	A7MS06	0.443	0.673	0.955	Flagellin
Vibhar_03173	A7MS08	0.425	0.705	1.000	Flagellin
Vibhar_03174	A7MS09	0.553	0.856	0.925	Flagellin
Vibhar_03143	A7MS30	1.052	0.953	0.455	CheY

a Determined by the peptide abundance after growth in the presence of NO divided by the abundance of the same peptide after growth in the presence of 0 nM NO.

**Table 2:** Bacterial strains used in this work.

Bacterial strains	Relevant characteristics	Ref.
*V. harveyi*		
WT	BB120, *V. harveyi* WT, ATCC BAA-1116	ATCC
Δ*luxO*	BB721, *luxO*:Tn5 ATCC 700106	ATCC
Δ*luxNS*	MM30, *luxN*::Cm, *luxS*::Tn5Kan, ATCC BAA-1120	ATCC
Δ*hnoX*	BB120 ΔVIBHAR_01911	[10]
*phnoX/*Δ*hnoX*	Δ*hnoX*, *phnoX*, Km^r^	[10]
