# Supplementary material for: A Nitric Oxide-Responsive Quorum Sensing Circuit in Vibrio harveyi Regulates Flagella Production and Biofilm Formation
Source: Int J Mol Sci. 2013 Aug 8;14(8):16473–84. doi: 10.3390/ijms140816473 (PMC3759921; doi:10.3390/ijms140816473)
Supplement: Supplementary file 1 [file ijms-14-16473-s001.pdf]

Supplementary Figure 1. *V. harveyi* growth curves of wild-type, Δ*hnoX*, and Δ*hnoX*/p*hnoX* in the presence of NO (50 nM NO, 100 nM NO, 500 nM NO) at 30 °C in AB medium. These data indicate that there is no significant delay in growth with less than 500 nM NO.

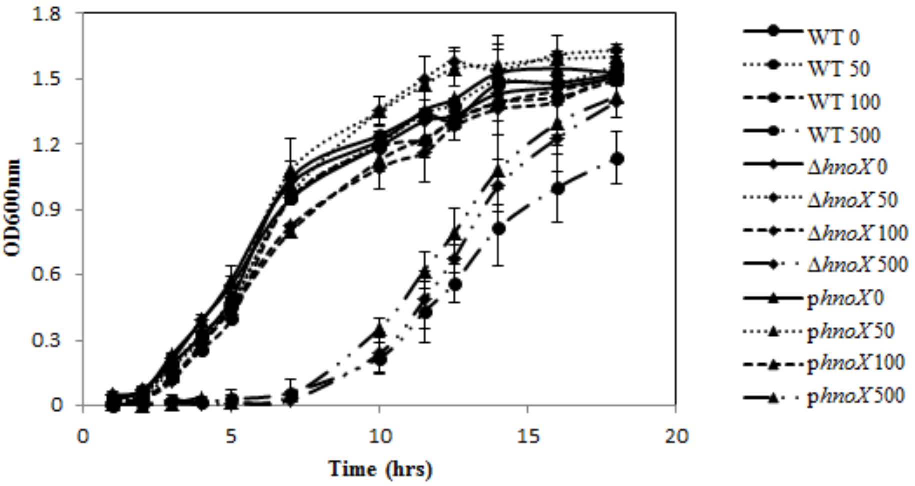

Supplementary Table 1. List of protein expression changes in *V. harveyi* as a function of NO. Peptides obtained after growth in the presence of 0, 50, 100, or 200 nM nitric oxide are represented by isobaric tags that produce signature ions at m/z 114, 115, 116 and 117 respectively. Standard deviations (S.D.) were obtained from readings of multiple significant peptides of the same protein. Peptide abundance is colored with down-regulated values in red and up-regulated values in green.

	Peptide abundance at (50/0) nM NO		Peptide abundance at (100/0) nM NO		Peptide abundance at (200/0) nM NO	
Protein Name	115/114	115/114 S.D.	116/114	116/114 S.D.	117/114	117/114 S.D.
adaA; methylphosphotriester-DNA alkyltransferase	0.197	0.040	0.142	0.031	0.327	0.014
uncharacterized protein	0.214	0.031	0.114	0.022	0.054	0.031
pyruvate dehydrogenase E1 component, alpha subunit	0.214	0.065	0.141	0.090	0.128	0.098
4-hydroxyphenylpyruvate dioxygenase	0.268	0.058	0.127	0.095	0.092	0.077
flagellin	0.365	0.091	0.625	0.201	0.813	0.228
flagellin	0.421	0.035	0.637	0.063	1.013	0.105
flagellin	0.425	0.102	0.705	0.104	1.000	0.065
flagellin	0.443	0.231	0.673	0.168	0.955	0.165
pknB; probable serine/threonine-protein kinase;	0.490	0.029	0.338	0.030	0.191	0.030
grxA; glutaredoxin 1	0.494	0.349	1.995	1.043	0.371	0.020
Type II secretory pathway, pseudopilin	0.535	0.189	0.450	0.186	0.393	0.382
Transcription antitermination protein nusG	0.580	0.038	0.527	0.020	0.402	0.064
OstA; OstA-like protein	0.589	0.129	0.927	0.110	0.464	0.112
Formimidoylglutamase	0.592	0.058	0.630	0.101	0.866	0.075
Enolase2	0.602	0.087	0.215	0.025	0.165	0.033
putative thioredoxin-like protein	0.606	0.101	0.955	0.056	0.308	0.262
gram_neg_porins	0.607	0.226	0.892	0.157	1.867	0.295
trpB; Tryptophan synthase beta chain	0.615	0.369	0.464	0.321	0.188	0.140
ATP-dependent zinc metalloprotease FtsH	0.629	0.307	0.619	0.299	0.859	0.341
L-allo-threonine aldolase	0.636	0.177	0.686	0.147	0.420	0.115
AcrR; Transcriptional regulator [Transcription]	0.637	0.091	1.472	0.138	3.794	1.061
Imidazolonepropionase	0.638	0.071	0.577	0.055	0.855	0.155
Amidinotransf; Amidinotransferase	0.660	0.126	0.681	0.165	0.367	0.079
Trypsin-like serine proteases, typically periplasmic, contain C-terminal PDZ domain	0.664	0.155	0.558	0.121	0.302	0.051
gamma-glutamyltransferase	0.665	0.135	0.842	0.104	0.599	0.096
Peptidase B	0.666	0.060	0.534	0.106	0.777	0.096
Urocanate hydratase	0.669	0.173	0.844	0.146	0.879	0.171
Integration host factor subunit alpha	0.672	0.125	0.933	0.119	2.036	0.417
Domain of unknown function	0.673	0.016	0.493	0.024	0.238	0.055

aroK; Shikimate kinase	0.674	0.247	0.624	0.375	0.352	0.290
ABC-type dipeptide transport system, periplasmic component	0.675	0.409	0.573	0.441	0.505	0.454
IgA1 protease	0.676	0.118	0.519	0.008	0.815	0.030
Salmonella repeat of unknown function (DUF823)	0.678	0.184	0.599	0.083	0.483	0.069
arginine N-succinyltransferase	0.687	0.094	0.653	0.085	0.805	0.087
fliY	0.688	0.488	0.467	0.271	0.374	0.404
nucleoid DNA-binding protein	0.707	0.252	1.125	0.344	2.666	1.107
inorganic pyrophosphatase	0.707	0.107	0.729	0.101	0.556	0.065
antioxidant, AhpC/TSA family protein	0.717	0.072	0.613	0.054	0.385	0.116
DNA-directed RNA polymerase sigma subunit	0.725	0.179	0.430	0.133	0.472	0.172
Single-stranded DNA-binding protein	0.727	0.122	1.215	0.130	1.361	0.132
entS; EntS/YbdA MFS transporter	0.732	0.038	0.579	0.107	0.496	0.061
OmpH; Outer membrane protein (OmpH-like)	0.733	0.035	0.775	0.168	0.662	0.331
50S ribosomal protein L3	0.736	0.121	0.976	0.201	1.343	0.273
leucine transcriptional activator	0.736	0.147	1.260	0.824	5.737	5.229
D-lactate dehydrogenase	0.742	0.105	0.803	0.112	0.786	0.166
Acyl-[acyl-carrier-protein]--UDP-N-acetylglucosamine O-acyl	0.743	0.041	0.758	0.035	0.666	0.253
Aspartate-semialdehyde dehydrogenase	0.744	0.111	0.614	0.066	0.608	0.115
coxB; cytochrome c oxidase	0.745	0.091	0.633	0.147	0.652	0.360
anti-RNA polymerase sigma 70 factor	0.747	0.054	0.608	0.117	0.347	0.082
FMN_red; NADPH-dependent FMN reductase	0.747	0.078	0.800	0.077	0.359	0.069
non supervised orthologous group	0.750	0.353	0.594	0.144	0.383	0.190
ATP-dependent Clp protease proteolytic subunit	0.752	0.153	0.678	0.122	0.707	0.134
Protein of unknown function (DUF2750)	0.755	0.287	0.873	0.335	0.747	0.076
Adenosine deaminase 1	0.761	0.034	0.945	0.038	0.764	0.055
UPF0312 protein; YceI; YceI-like domain	0.762	0.188	0.772	0.235	0.507	0.179
D-3-phosphoglycerate dehydrogenase	0.762	0.157	0.670	0.179	2.698	0.752
Protein of unknown function (DUF1451)	0.766	0.169	0.716	0.181	0.288	0.093
ABC-type oligopeptide transport system, periplasmic component	0.767	0.178	0.577	0.180	0.592	0.152
aminopeptidase N	0.776	0.188	0.614	0.138	1.090	0.342
Peptide chain release factor 3	0.784	0.018	0.648	0.044	0.903	0.248
Protein of unknown function	0.784	0.152	0.763	0.227	0.340	0.084
DNA-binding transcriptional regulator Crl	0.786	0.177	0.656	0.143	0.381	0.103
3,4-dihydroxy-2-butanone 4-phosphate synthase	0.792	0.186	0.856	0.110	0.964	0.172
Chaperone protein hscA homolog	0.795	0.158	0.970	0.172	0.981	0.177
ABC amino acid transporter periplasmic ligand binding protein	0.795	0.175	0.660	0.007	0.614	0.046
chemotaxis protein CheX	0.797	0.132	0.991	0.058	0.596	0.094
Putative translation initiation inhibitor, yjgF family	0.804	0.159	0.929	0.194	0.573	0.150
30S ribosomal protein S11	0.809	0.448	0.798	0.507	1.481	1.002
ribosome-associated protein Y	0.810	0.173	1.166	0.451	1.515	0.987
3-isopropylmalate dehydrogenase	0.810	0.045	0.728	0.078	0.456	0.079
OmpR; osmolarity response regulator	0.811	0.320	0.740	0.316	0.518	0.226
aroC; Chorismate synthase	0.813	0.056	0.744	0.041	0.464	0.042

greA2; Transcription elongation factor	0.814	0.132	1.041	0.270	0.595	0.252
glycerol-3-phosphate dehydrogenase	0.819	0.027	0.594	0.044	1.403	0.071
Fe-S_biosyn; Iron-sulphur cluster biosynthesis	0.824	0.086	0.971	0.081	0.557	0.095
Arginine deiminase	0.824	0.081	0.929	0.214	1.109	0.375
GAF domain-containing protein	0.824	0.094	0.777	0.134	0.558	0.146
Asparaginyl-tRNA synthetase	0.827	0.139	0.562	0.133	0.849	0.202
UPF0502 protein	0.827	0.177	1.455	0.353	2.150	0.559
Phosphate-starvation-inducible E	0.828	0.167	0.937	0.090	0.722	0.061
sthA; Soluble pyridine nucleotide transhydrogenase	0.828	0.139	0.581	0.092	0.493	0.107
ferric uptake regulator	0.829	0.114	1.066	0.085	0.764	0.092
Probable cytosol aminopeptidase	0.830	0.149	1.441	0.250	4.428	1.101
Protein of unknown function (DUF1499)	0.834	0.062	0.911	0.071	0.468	0.055
50S ribosomal protein	0.836	0.304	0.772	0.359	1.408	0.762
Glutaminyl-tRNA synthetase	0.839	0.245	0.930	0.277	0.911	0.248
trimethylamine-N-oxide reductase	0.841	0.054	1.081	0.155	0.519	0.097
50S ribosomal protein L6	0.846	0.131	1.037	0.228	1.195	0.197
DNA-binding transcriptional regulator TorR	0.851	0.129	1.842	0.302	6.595	1.184
deoC; Deoxyribose-phosphate aldolase	0.858	0.076	0.667	0.117	0.551	0.167
Dihydrodipicolinate reductase	0.860	0.109	0.805	0.103	0.623	0.110
bifunctional phosphoribosylaminoimidazolecarboxamide formyltransferase/IMP cyclohydrolase, Bifunctional purine biosynthesis protein purH	0.860	0.097	0.669	0.113	0.263	0.034
ATP synthase epsilon chain 1	0.862	0.153	0.567	0.251	0.624	0.585
Protein-disulfide isomerase	0.863	0.097	1.086	0.173	1.001	0.166
Nitrogen regulatory protein PII	0.864	0.033	1.026	0.087	0.717	0.016
LUXS S-ribosylhomocysteine lyase	0.866	0.137	1.091	0.221	0.652	0.101
4,5-dioxygenase	0.868	0.091	0.733	0.211	0.422	0.028
fatty acid reductase	0.876	0.299	0.892	0.335	1.279	0.322
	0.881	0.099	1.449	0.093	0.813	0.428
	0.881	0.071	0.990	0.105	1.358	0.140
oxidoreductase	0.883	0.120	0.892	0.017	0.851	0.079
N-succinylglutamate 5-semialdehyde dehydrogenase	0.883	0.103	0.426	0.068	0.286	0.076
Protein of unknown function (DUF339)	0.884	0.115	0.969	0.091	0.557	0.074
bifunctional 2',3'-cyclic nucleotide 2'-phosphodiesterase/3'-nucleotidase periplasmic precursor protein	0.885	0.156	0.962	0.111	0.892	0.206
argD; bifunctional N-succinyldiaminopimelate-aminotransferase/acetylornithine transaminase protein	0.885	0.106	0.519	0.137	0.471	0.137
Acetyl-coenzyme A synthetase 2	0.886	0.047	0.477	0.063	0.574	0.032
phosphoheptose isomerase	0.887	0.165	0.999	0.158	0.680	0.161
Gamma-glutamyl phosphate reductase	0.888	0.128	0.763	0.092	1.165	0.126
phosphomannomutase	0.889	0.048	0.394	0.027	0.238	0.027
carboxy-terminal protease	0.892	0.166	0.772	0.204	0.928	0.292
ribonuclease E	0.895	0.170	1.008	0.123	1.578	0.722
ATP-dependent Clp protease ATP-binding subunit	0.896	0.094	0.644	0.050	0.304	0.100
Dihydroorotase	0.897	0.183	1.360	0.349	0.856	0.177

Serine proteases of the peptidase family S9A	0.910	0.127	1.026	0.168	0.913	0.188
ABC-type Zn2+ transport system, periplasmic component/surface adhesin	0.914	0.372	0.895	0.395	0.600	0.086
Cell division protein zapB	0.915	0.140	1.093	0.143	0.567	0.091
Peptide deformylase OS	0.916	0.104	0.984	0.070	0.762	0.124
Serine hydroxymethyltransferase	0.917	0.134	1.007	0.180	0.493	0.095
50S ribosomal protein L18	0.919	0.196	0.972	0.340	1.299	0.489
Cysteine desulfurase	0.922	0.231	0.835	0.372	1.203	0.583
choloylglycine hydrolase	0.924	0.086	1.284	0.098	1.066	0.104
Spermidine/putrescine-binding periplasmic protein	0.924	0.079	1.070	0.060	0.716	0.002
Uracil phosphoribosyltransferase	0.925	0.150	1.108	0.275	0.530	0.086
Purine nucleoside phosphorylase deoD-type 2	0.926	0.223	0.794	0.146	0.744	0.147
UPF0319 protein	0.926	0.175	1.113	0.208	0.666	0.178
50S ribosomal protein L13	0.928	0.204	1.001	0.182	1.577	0.254
DNA-binding protein H-NS	0.929	0.706	1.558	1.742	2.513	3.902
30S ribosomal protein S6	0.929	0.161	1.134	0.164	1.307	0.215
Sporulation control protein	0.930	0.154	0.789	0.032	1.100	0.092
fdx; ferredoxin, 2Fe-2S	0.930	0.187	1.133	0.362	0.641	0.228
antioxidant, AhpC/Tsa family	0.934	0.398	1.032	0.249	0.852	0.306
phosphoglucomutase	0.937	0.198	0.922	0.129	0.292	0.083
Fatty acid metabolism regulator protein	0.937	0.105	1.133	0.177	0.623	0.083
Elongation factor Tu	0.937	0.499	0.907	0.307	0.507	0.211
uncharacterized protein	0.938	0.116	0.921	0.120	0.884	0.139
50S ribosomal protein L7/L12	0.940	0.413	1.388	0.710	0.900	0.501
50S ribosomal protein L24	0.940	0.355	1.220	0.110	1.239	0.357
Protein of unknown function (DUF541)	0.941	0.045	0.896	0.123	1.171	0.082
30S ribosomal protein S14	0.942	0.337	0.787	0.272	1.335	0.720
uncharacterized protein	0.944	0.078	1.151	0.138	0.624	0.063
glyceraldehyde 3-phosphate dehydrogenase	0.944	0.823	0.679	0.161	0.887	0.318
DNA-directed RNA polymerase subunit beta	0.944	0.178	0.992	0.178	3.305	0.701
ATP synthase subunit delta 1	0.944	0.150	1.008	0.303	0.678	0.137
HlyU; Transcriptional activator HlyU	0.946	0.148	0.925	0.102	0.500	0.067
Porphobilinogen deaminase	0.946	0.093	0.977	0.248	0.604	0.138
trans-2-enoyl-CoA reductase	0.947	0.122	0.821	0.079	0.606	0.153
ABC-type Fe3+ transport system, periplasmic component	0.947	0.451	0.767	0.124	0.852	0.135
ATP-dependent protease	0.949	0.163	0.967	0.271	3.234	1.233
acetyl-CoA synthetase	0.949	0.126	0.507	0.101	0.600	0.097
Phosphoglucosamine mutase	0.950	0.459	0.848	0.524	1.217	1.147
phosphoribosylformylglycinamidine synthase	0.952	0.116	0.874	0.179	1.389	0.419
acetyl-CoA carboxylase biotin carboxylase subunit	0.954	0.225	0.761	0.166	0.699	0.153
Putative heme iron utilization protein	0.955	0.285	0.836	0.261	0.449	0.198
Amidophosphoribosyltransferase	0.956	0.131	1.049	0.133	2.584	0.361
50S ribosomal protein L19	0.958	0.055	0.900	0.077	1.333	0.146
Manganese superoxide dismutase	0.962	0.043	0.249	0.045	0.104	0.043
30S ribosomal protein S16	0.963	0.149	1.135	0.246	1.126	0.179
glycerophosphodiester phosphodiesterase	0.963	0.430	1.016	0.457	0.905	0.356
Probable Fe(2+)-trafficking protein	0.964	0.457	0.927	0.099	0.504	0.147

Pyridoxine 5'-phosphate synthase	0.966	0.125	1.000	0.121	0.958	0.136
Peptidyl-prolyl cis-trans isomerase	0.970	0.085	1.107	0.086	0.822	0.168
Peptidyl-prolyl cis-trans isomerase	0.971	0.207	1.133	0.332	0.497	0.142
phosphoribosylamine--glycine ligase	0.972	0.111	0.975	0.107	0.367	0.090
maltose ABC transporter periplasmic protein	0.973	0.248	1.002	0.229	0.821	0.179
peptidase	0.973	0.087	0.913	0.118	1.325	0.315
Iron-sulfur cluster insertion protein erpA	0.977	0.097	1.218	0.223	0.880	0.041
30S ribosomal protein S3	0.979	0.091	0.833	0.111	2.479	0.153
Peptide chain release factor 1	0.979	0.312	1.010	0.142	0.734	0.111
pknB; probable serine/threonine-protein kinase	0.979	0.730	0.885	0.079	0.538	0.117
30S ribosomal protein S4	0.982	0.172	1.095	0.141	1.882	0.393
Uroporphyrinogen decarboxylase	0.982	0.210	1.170	0.118	1.482	0.280
nitroreductase A	0.982	0.059	0.870	0.342	0.614	0.029
Nitroreductase	0.984	0.107	1.283	0.096	1.017	0.151
50S ribosomal protein L4	0.985	0.355	1.082	0.520	1.752	0.818
fumC; fumarate hydratase	0.985	0.094	0.348	0.045	0.127	0.039
putative manganese-dependent inorganic pyrophosphatase	0.985	0.220	1.155	0.186	0.878	0.157
DNA-directed RNA polymerase subunit beta	0.986	0.202	0.970	0.199	3.089	0.840
putative type I restriction-modification system, methyltransferase subunit	0.987	0.078	1.096	0.125	0.913	0.097
50S ribosomal protein L5	0.987	0.087	1.023	0.090	2.237	0.357
Glycine dehydrogenase [decarboxylating]	0.989	0.198	1.260	0.261	3.787	1.124
oxygen-independent coproporphyrinogen III oxidase	0.990	0.100	0.819	0.085	0.527	0.068
adenylate cyclase	0.991	0.159	0.736	0.102	0.430	0.030
Acyl carrier protein	0.992	0.202	1.247	0.287	0.602	0.269
Peptidyl-prolyl cis-trans isomerase	0.994	0.117	0.979	0.163	0.534	0.167
PKCI_related; Protein Kinase C Interacting protein related (PKCI): PKCI and related proteins belong to the ubiquitous HIT family of hydrolases that act on alpha-phosphates of ribonucleotides	0.995	0.116	0.984	0.137	0.401	0.063
3-hydroxydecanoyl-[acyl-carrier-protein] dehydratase	0.995	0.187	1.313	0.233	2.303	0.648
50S ribosomal protein L16	0.995	0.088	1.102	0.108	2.233	0.260
3'(2'),5'-bisphosphate nucleotidase	0.995	0.153	0.776	0.024	0.737	0.021
30S ribosomal protein	1.000	0.310	1.078	0.194	1.424	0.251
2-dehydro-3-deoxyphosphooctonate aldolase	1.000	0.114	1.174	0.105	1.017	0.124
isoprenoid biosynthesis protein with amidotransferase-like domain	1.001	0.256	0.906	0.137	0.601	0.545
Glycine cleavage system H protein	1.001	0.129	1.628	0.307	0.850	0.122
Bifunctional protein folD	1.003	0.110	1.342	0.198	0.885	0.104
Phosphoglycerate kinase	1.003	0.430	0.934	0.499	0.411	0.340
50S ribosomal protein L21	1.004	0.418	1.076	0.196	1.341	0.358
peptidase	1.006	0.396	0.712	0.111	0.855	0.130
Phosphate transport regulator (distant homolog of PhoU)~	1.008	0.110	0.823	0.085	0.401	0.066
30S ribosomal protein S9	1.013	0.160	1.059	0.178	2.393	0.458
aromatic amino acid aminotransferase	1.014	0.067	0.641	0.273	0.886	0.139
alkanal monooxygenase alpha chain	1.014	0.312	0.923	0.253	1.063	0.301

Aspartyl-tRNA synthetase	1.014	0.137	0.809	0.129	0.978	0.161
Isoleucyl-tRNA synthetase	1.014	0.215	1.059	0.249	1.592	0.357
nucleotide sugar dehydrogenase	1.017	0.223	0.597	0.034	0.266	0.026
50S ribosomal protein L14	1.017	0.287	1.387	0.478	2.282	0.986
scaffold protein	1.021	0.351	1.396	0.572	0.940	0.377
HTH-type transcriptional repressor purR	1.021	0.339	0.949	0.350	1.452	0.843
Adenylate kinase	1.021	0.163	1.166	0.228	0.733	0.171
3-methyl-2-oxobutanoate hydroxymethyltransferase	1.021	0.324	1.177	0.241	1.422	0.312
Triosephosphate isomerase	1.023	0.813	1.038	0.210	0.726	0.169
DNA polymerase I	1.023	0.041	0.811	0.048	2.026	0.618
50S ribosomal protein	1.024	0.139	1.161	0.126	1.394	0.129
Protein of unknown function (DUF1244)	1.024	0.102	1.280	0.075	0.824	0.105
30S ribosomal protein S21	1.025	0.085	1.067	0.078	1.348	0.161
Ribonucleoside-diphosphate reductase	1.028	0.010	0.775	0.053	1.167	0.053
coxB; cytochrome c oxidase, subunit II	1.028	0.537	1.575	0.524	1.430	1.005
Ribulose-phosphate 3-epimerase	1.029	0.163	1.184	0.235	1.012	0.184
adenylosuccinate synthetase	1.029	0.462	0.567	0.248	0.264	0.116
Thiol:disulfide interchange protein DsbA	1.032	0.115	1.076	0.127	0.640	0.179
Fe/S biogenesis protein nfuA	1.032	0.063	1.799	0.171	0.645	0.059
6,7-dimethyl-8-ribityllumazine synthase	1.034	0.126	1.058	0.111	1.185	0.126
thiamine transporter substrate binding subunit	1.035	0.341	0.783	0.086	0.920	0.182
plasmid pVIBHAR	1.036	0.247	0.944	0.314	0.248	0.076
signal recognition particle GTPase	1.037	0.169	1.168	0.226	0.955	0.282
Erythronate-4-phosphate dehydrogenase	1.040	0.105	0.901	0.055	1.373	0.042
carboxypeptidase	1.040	0.166	0.729	0.111	0.972	0.233
surA; survival protein SurA	1.041	0.134	1.187	0.193	0.897	0.152
Arginyl-tRNA synthetase	1.041	0.139	0.966	0.133	1.510	0.208
50S ribosomal protein L9	1.042	0.300	1.131	0.175	1.465	0.284
uncharacterized protein	1.042	0.006	1.199	0.072	0.761	0.267
aromatic amino acid aminotransferase	1.043	0.175	0.968	0.115	1.683	0.317
GTP cyclohydrolase 1	1.043	0.060	1.207	0.154	0.981	0.103
Succinylglutamate desuccinylase	1.047	0.127	0.830	0.125	1.520	0.359
UPF0265 protein	1.047	0.111	1.057	0.112	0.511	0.064
AcrR; Transcriptional regulator [Transcription]	1.050	0.516	1.278	0.361	3.873	1.628
prolyl oligopeptidase	1.051	0.185	1.052	0.181	0.937	0.153
cheY; chemotaxis protein CheY	1.052	0.074	0.953	0.052	0.455	0.030
Oligoribonuclease	1.053	0.049	0.995	0.092	0.600	0.091
Phosphoribosylglycinamide formyltransferase 2	1.055	0.246	0.953	0.207	0.953	0.177
pyruvate-formate lyase	1.055	0.220	1.175	0.208	2.547	0.760
ATP-dependent protease La	1.057	0.257	0.861	0.177	0.897	0.304
3,4-dihydroxy-2-butanone 4-phosphate synthase	1.057	0.291	1.021	0.392	0.614	0.187
2-oxoglutarate dehydrogenase E1 component	1.059	0.167	0.930	0.156	2.711	0.558
50S ribosomal protein L17	1.061	0.192	1.222	0.254	1.228	0.201
hypothetical protein	1.062	0.080	1.026	0.249	2.944	0.549
GMP synthase [glutamine-hydrolyzing]	1.064	0.046	1.225	0.102	3.042	0.406
tryptophanyl-tRNA synthetase	1.066	0.046	1.081	0.198	1.157	0.376

predicted iron-dependent peroxidase	1.068	0.240	1.176	0.140	1.323	0.858
50S ribosomal protein L11	1.068	0.574	1.090	0.188	1.310	0.479
cytochrome b562	1.069	0.047	1.294	0.174	0.854	0.128
30S ribosomal protein S18	1.072	0.491	0.941	0.196	1.863	0.620
Tryptophan synthase alpha chain	1.074	0.075	1.067	0.201	0.787	0.206
Glutamate-1-semialdehyde 2,1-aminomutase	1.075	0.363	0.855	0.313	0.338	0.122
glutamate dehydrogenase	1.076	0.371	0.676	0.129	1.041	0.322
Carbamoyl-phosphate synthase large chain	1.078	0.133	1.101	0.134	1.371	0.195
33 kDa chaperonin	1.079	0.210	1.353	0.206	1.149	0.243
30S ribosomal protein S19	1.079	0.179	1.361	0.248	1.338	0.048
ADP-ribose diphosphatase NudE	1.079	0.177	0.898	0.124	0.884	0.141
Peptidase_M14-like_4; A functionally uncharacterized subgroup of the M14 family of metallocarboxypeptidases (MCPs).	1.080	0.295	1.212	0.451	0.840	0.224
thioredoxin	1.081	0.143	1.195	0.108	0.769	0.096
Pyruvate dehydrogenase E1 component	1.082	0.220	1.055	0.191	3.234	0.918
Pyruvate kinase	1.083	0.177	0.951	0.151	1.645	0.273
pyrazinamidase/nicotinamidase	1.086	0.337	1.198	0.350	0.889	0.100
iron-containing alcohol dehydrogenase	1.086	0.292	1.485	0.164	0.492	0.137
Phosphopantetheine attachment site	1.087	0.243	1.024	0.163	0.548	0.134
Peptidyl-prolyl cis-trans isomerase	1.087	0.298	1.215	0.345	0.766	0.287
cb-type cytochrome oxidase subunit III	1.088	0.098	1.693	0.379	0.677	0.091
30S ribosomal protein S8	1.090	0.098	1.047	0.093	2.114	0.216
Elongation factor P	1.090	0.216	1.179	0.329	1.108	0.301
Polyribonucleotide nucleotidyltransferase	1.090	0.591	1.084	0.702	1.998	1.740
heat shock protein	1.092	0.156	1.540	0.293	3.063	0.741
Ribosome-recycling factor	1.092	0.207	1.200	0.271	0.789	0.269
UPF0149 protein	1.093	0.121	1.067	0.101	0.582	0.118
Zn-dependent oligopeptidase	1.093	0.198	0.885	0.111	1.691	0.374
Seryl-tRNA synthetase	1.093	0.168	1.250	0.254	1.049	0.192
50S ribosomal protein L2	1.094	0.159	1.160	0.104	2.053	0.456
succinate dehydrogenase iron-sulfur subunit	1.095	0.222	1.245	0.164	3.122	0.453
DNA gyrase subunit B	1.096	0.121	0.806	0.049	1.117	0.247
Threonyl-tRNA synthetase	1.097	0.127	0.889	0.092	1.209	0.151
50S ribosomal protein L28	1.099	0.082	1.110	0.124	1.739	0.219
phosphoenolpyruvate-protein phosphotransferase	1.099	0.128	1.110	0.222	1.235	0.306
phosphoribosylaminoimidazole carboxylase ATPase subunit	1.102	0.097	1.152	0.147	1.231	0.155
UPF0133 protein	1.103	0.271	1.253	0.358	0.929	0.338
Fructose-1,6-bisphosphatase class 1	1.105	0.190	1.118	0.213	1.340	0.291
Chaperone protein dnaJ	1.106	0.067	0.896	0.013	0.834	0.036
Translation initiation factor IF-2	1.107	0.356	0.857	0.256	1.236	0.502
50S ribosomal protein L32	1.108	0.405	1.529	0.670	1.540	0.642
Phosphoserine aminotransferase	1.108	0.037	0.911	0.142	0.751	0.141
NAD-dependent malic enzyme	1.110	0.184	1.012	0.166	3.261	0.773
50S ribosomal protein L22	1.110	0.702	0.993	0.404	1.432	0.680
50S ribosomal protein L10	1.110	0.178	1.206	0.257	1.625	0.418
Aspartate carbamoyltransferase regulatory chain	1.110	0.030	1.105	0.009	1.554	0.106

carbonic anhydrase	1.115	0.207	1.210	0.205	0.845	0.193
UDP-N-acetylglucosamine 2-epimerase	1.116	0.190	1.075	0.194	0.284	0.076
Protein-export protein secB	1.117	0.166	1.171	0.151	0.909	0.340
Chaperone protein dnaK	1.118	0.647	1.289	0.501	0.898	0.358
peroxiredoxin	1.118	0.102	0.981	0.083	0.514	0.175
RNA polymerase sigma factor	1.119	0.205	0.981	0.127	2.440	0.687
Peptidase_M75; Imelysin	1.120	0.164	1.148	0.233	0.858	0.263
Trigger factor	1.120	0.725	1.674	1.350	1.988	1.876
zinc-carboxypeptidase	1.120	0.082	1.059	0.078	1.198	0.097
asparagine synthetase B	1.121	0.254	0.690	0.083	0.367	0.119
archaeal Glu-tRNA(Gln) amidotransferase, subunit E containing GAD domain	1.122	0.136	1.374	0.224	0.786	0.161
Leucyl-tRNA synthetase	1.122	0.223	1.203	0.202	1.791	0.311
ADP-L-glycero-D-manno-heptose-6-epimerase	1.122	0.097	0.865	0.129	1.016	0.172
phenylalanyl-tRNA synthetase subunit beta	1.122	0.156	1.062	0.121	1.973	0.275
Catalase	1.123	0.145	1.547	0.269	1.478	0.272
DNA-directed RNA polymerase subunit alpha	1.124	0.224	1.207	0.251	2.076	0.422
Transcriptional regulators of sugar metabolism	1.128	0.023	0.859	0.133	1.054	0.023
putative alcohol dehydrogenase	1.130	0.110	0.739	0.112	0.662	0.062
50S ribosomal protein L1	1.130	0.286	1.248	0.288	1.344	0.276
glutathione synthetase	1.132	0.228	1.133	0.287	1.166	0.426
Histidyl-tRNA synthetase	1.134	0.270	1.086	0.253	1.925	1.040
Xaa-Pro aminopeptidase	1.135	0.176	0.900	0.099	1.739	0.261
Elongation factor G 2	1.135	0.310	0.998	0.187	1.467	0.319
malate oxidoreductase	1.137	0.155	1.211	0.139	1.293	0.199
FMN reductase	1.137	0.140	1.002	0.131	0.820	0.108
6-phosphofructokinase	1.141	0.161	0.961	0.083	1.223	0.013
Transaldolase	1.142	0.261	1.034	0.254	0.986	0.348
Delta-aminolevulinic acid dehydratase	1.145	0.178	1.092	0.232	0.993	0.271
putative nucleotide-binding protein	1.146	0.376	1.422	0.427	1.178	0.232
dihydrodipicolinate synthase	1.147	0.129	1.190	0.127	0.289	0.051
UPF0176 protein	1.149	0.203	1.312	0.300	0.662	0.095
Diaminopimelate decarboxylase	1.150	0.139	1.001	0.107	1.232	0.143
beta-hexosaminidase	1.151	0.077	0.961	0.084	1.484	0.302
Lysyl-tRNA synthetase	1.151	0.133	0.982	0.196	0.633	0.110
30S ribosomal protein S5	1.152	0.234	1.233	0.300	2.146	0.461
UDP-N-acetylglucosamine 1-carboxyvinyltransferase	1.157	0.519	0.908	0.507	1.534	0.732
positive response regulator for pho regulon	1.157	0.069	1.053	0.077	0.942	0.096
Sulfite reductase [NADPH] hemoprotein beta-component	1.160	0.257	1.772	0.501	1.603	0.430
phosphoenolpyruvate synthase	1.161	0.212	1.186	0.220	2.395	0.592
3-oxoacyl-[acyl-carrier-protein] synthase 2	1.163	0.074	1.013	0.113	0.683	0.122
alkanal monooxygenase beta chain	1.164	0.292	0.930	0.177	1.475	0.348
TelA; Toxic anion resistance protein (TelA)	1.165	0.250	1.368	0.266	1.103	0.334
ATP-dependent protease	1.168	0.190	1.101	0.179	1.418	0.217
60 kDa chaperonin	1.169	0.269	1.030	0.219	1.319	0.303
Enolase 1	1.173	0.358	1.238	0.292	0.665	0.241
phosphoribosylaminoimidazole carboxylase catalytic subunit	1.173	0.209	1.252	0.296	1.597	0.205

general secretory pathway protein E	1.175	0.103	1.145	0.130	1.494	0.133
Phosphoenolpyruvate carboxykinase [ATP]	1.176	0.448	0.946	0.271	1.453	0.402
30S ribosomal protein S7	1.178	0.276	1.111	0.181	1.971	0.439
Transcription termination factor Rho	1.185	0.429	0.903	0.283	0.857	0.499
Aspartate carbamoyltransferase	1.187	0.235	0.977	0.210	2.000	0.673
stringent starvation protein A	1.187	0.109	0.931	0.071	1.909	0.214
Alanyl-tRNA synthetase	1.193	0.325	1.321	0.529	1.528	0.481
Succinyl-CoA ligase [ADP-forming] subunit beta	1.193	0.451	1.336	0.295	0.997	0.260
Glycerol kinase	1.195	0.121	0.952	0.140	2.899	0.373
DNA polymerase III subunit beta	1.198	0.206	1.450	0.211	1.762	0.175
ribosome-associated protein Y	1.198	0.112	1.176	0.067	1.465	0.095
putative ABC transporter ATP-binding protein	1.200	0.257	1.084	0.146	0.498	0.204
Alanine dehydrogenase	1.203	0.221	0.848	0.138	1.754	0.310
Glutamyl-tRNA synthetase	1.205	0.112	0.981	0.078	0.713	0.138
2,3-bisphosphoglycerate-independent phosphoglycerate mutase	1.206	0.586	0.969	0.311	0.705	0.190
phosphocarrier protein PtsH	1.208	0.139	1.399	0.025	0.678	0.019
Inosine-5'-monophosphate dehydrogenase	1.208	0.503	0.986	0.526	0.449	0.171
30S ribosomal protein S2	1.210	0.350	1.105	0.365	1.668	0.839
transcription elongation factor NusA	1.211	0.281	1.106	0.290	1.205	0.342
Elongation factor G 1	1.211	0.094	1.121	0.163	1.152	0.161
Valyl-tRNA synthetase	1.215	0.182	1.321	0.206	1.258	0.189
EntF; Non-ribosomal peptide synthetase modules and related proteins	1.216	0.111	0.582	0.081	0.184	0.035
ribonucleotide-diphosphate reductase subunit beta	1.216	0.171	1.340	0.159	1.929	0.979
Glucose-6-phosphate 1-dehydrogenase	1.217	0.217	1.071	0.200	2.274	0.639
transketolase	1.218	0.285	1.067	0.262	1.611	0.397
ketol-acid reductoisomerase	1.218	0.354	0.980	0.198	1.115	0.251
Glutamine synthetase	1.222	0.444	1.014	0.218	1.508	0.246
GTP-binding protein engA	1.224	0.376	0.828	0.309	0.854	0.328
Thioredoxin reductase	1.225	0.668	1.041	0.079	0.951	0.130
Glutamate--cysteine ligase	1.225	0.196	1.135	0.195	1.124	0.293
Glucose-6-phosphate isomerase	1.226	0.198	1.147	0.214	1.508	0.467
bifunctional proline dehydrogenase/pyrroline-5-carboxylate dehydrogenase	1.226	0.252	1.060	0.143	1.696	0.380
succinate dehydrogenase flavoprotein subunit	1.229	0.230	1.185	0.208	1.861	0.577
Dihydrolipoyl dehydrogenase	1.229	0.246	1.468	0.337	2.191	0.647
Isocitrate dehydrogenase [NADP]	1.229	0.510	0.932	0.270	0.562	0.179
glyS; Glycyl-tRNA synthetase beta subunit	1.232	0.272	1.061	0.265	0.557	0.115
ATP synthase gamma chain	1.232	0.365	1.039	0.233	1.721	0.708
Cytidylate kinase	1.236	0.284	1.439	0.154	0.874	0.145
Carbon storage regulator homolog	1.236	0.144	1.189	0.461	0.990	0.032
Putative Mg2+ and Co2+ transporter	1.238	0.271	1.435	0.317	1.247	0.351
ABC amino acid transporter periplasmic component	1.238	0.225	1.750	0.392	1.557	0.336
DUF1887; Protein of unknown function,	1.239	0.147	1.194	0.209	2.318	0.370
Serine hydroxymethyltransferase	1.241	0.231	0.869	0.166	0.348	0.205
PTS system glucose-specific transporter	1.248	0.963	1.100	0.581	0.609	0.240

subunit						
4-hydroxy-3-methylbut-2-en-1-yl diphosphate synthase	1.250	0.045	1.193	0.068	1.108	0.086
Spermidine/putrescine-binding periplasmic protein	1.254	0.849	0.803	0.268	0.579	0.349
50S ribosomal protein L23	1.255	0.400	1.334	0.462	1.671	0.556
glucosamine--fructose-6-phosphate aminotransferase	1.257	0.173	1.217	0.179	1.414	0.214
uncharacterized protein	1.258	0.291	1.748	0.314	0.893	0.218
Aminomethyltransferase	1.259	0.423	1.402	0.473	1.493	0.536
uncharacterized protein	1.264	0.142	1.626	0.193	0.648	0.146
malonyl CoA-acyl carrier protein transacylase	1.266	0.005	1.202	0.015	0.590	0.004
Phosphoribosylaminoimidazole-succinocarboxamide synthase	1.269	0.234	0.881	0.143	0.326	0.112
Protein translocase subunit secA	1.269	0.358	0.791	0.157	1.004	0.236
YceI; YceI-like domain	1.270	0.477	1.323	0.117	0.955	0.212
prfB; protein chain release factor B	1.271	0.262	1.212	0.224	0.504	0.126
transketolase	1.272	0.184	1.043	0.172	1.621	0.279
ATP synthase subunit alpha 1	1.272	0.377	0.956	0.205	1.944	0.568
6-phosphogluconate dehydrogenase, decarboxylating	1.272	0.286	1.268	0.389	1.685	0.431
Prolyl-tRNA synthetase	1.273	0.425	1.090	0.340	1.554	0.544
dihydrolipoamide acetyltransferase	1.277	0.275	1.301	0.306	1.920	0.489
glutamate decarboxylase	1.277	0.241	1.225	0.218	1.254	0.340
hypoxanTHIne ribosyl transferase	1.278	0.092	1.195	0.098	1.080	0.099
Ubiquinol-cytochrome c reductase iron-sulfur subunit	1.285	0.255	2.094	0.141	1.566	0.354
typA, bipA, yihK, yjhK; putative GTP-binding factor	1.285	0.636	1.755	0.933	4.672	2.848
ferredoxin/oxidoreductase	1.286	0.280	0.903	0.134	0.949	0.052
carbamoyl phosphate synthase small subunit	1.290	0.296	1.387	0.368	0.924	0.248
ATP-dependent hsl protease ATP-binding subunit	1.291	0.422	0.923	0.223	0.837	0.237
Methionyl-tRNA synthetase	1.291	0.415	1.291	0.400	2.579	0.897
6-phosphogluconolactonase/Glucosamine-6-phosphate isomerase/deaminase	1.299	0.095	1.122	0.022	1.396	0.089
Aconitate hydratase 2	1.300	0.279	1.340	0.300	1.450	0.361
cheW; CheW positive regulator of CheA protein activity	1.300	0.135	1.251	0.151	1.158	0.139
ubiquinone/menaquinone biosynthesis methyltransferase	1.301	0.323	0.943	0.171	0.574	0.169
2,3,4,5-tetrahydropyridine-2-carboxylate N-succinyltransferase	1.305	0.197	0.825	0.067	0.820	0.180
cysteinyl-tRNA synthetase	1.308	0.175	1.193	0.121	1.946	0.319
pterin-4-alpha-carbinolamine dehydratase	1.309	0.180	1.693	0.362	1.422	0.180
Tyrosyl-tRNA synthetase	1.313	0.185	1.123	0.201	0.909	0.050
30S ribosomal protein S1	1.322	0.278	1.531	0.389	1.958	0.517
fructose-bisphosphate aldolase	1.323	0.850	0.931	0.344	1.204	0.579
UPF0082 protein	1.323	0.408	1.375	0.445	0.949	0.184
Phosphatidylserine/phosphatidylglycerophosphate/cardiolipin synthases and related enzymes [Lipid metabolism	1.325	0.116	1.250	0.030	3.161	0.291

ferritin	1.332	0.582	1.114	0.340	1.106	0.666
acetyl-CoA acetyltransferase	1.341	0.527	0.886	0.394	0.430	0.242
Phosphate acetyltransferase	1.343	0.249	0.888	0.235	0.718	0.196
adenylosuccinate lyase	1.343	0.259	1.185	0.278	2.926	0.893
metK; S-adenosylmethionine synthase	1.345	0.093	0.979	0.064	0.492	0.063
nitrate/sulfonate/bicarbonate transport systems, periplasmic components	1.345	0.205	1.525	0.321	1.371	0.286
Ribose-5-phosphate isomerase A	1.348	0.722	1.140	0.144	1.164	0.179
Pantothenate synthetase	1.380	0.166	1.161	0.157	1.606	0.310
AckA Acetate kinase	1.380	0.091	0.921	0.095	0.542	0.169
CTP synthase	1.383	0.380	1.142	0.367	0.631	0.289
Elongation factor Ts	1.387	0.755	1.467	0.439	1.430	0.500
Protein of unknown function (DUF3549)	1.392	0.198	1.072	0.041	0.440	0.008
Protein grpE	1.396	0.577	1.705	0.454	1.375	0.430
clpB; heat shock protein	1.399	0.542	1.093	0.669	0.613	0.352
DNA gyrase subunit A	1.403	0.490	1.255	0.602	3.725	1.903
Orotidine 5'-phosphate decarboxylase	1.411	0.162	1.215	0.095	1.190	0.068
inositol monophosphate family protein	1.412	0.563	1.986	1.243	2.292	1.229
Adenine phosphoribosyltransferase	1.426	0.121	1.238	0.202	0.343	0.079
ATP synthase subunit beta 1	1.429	0.406	1.060	0.238	2.193	0.499
Thiamine-phosphate pyrophosphorylase	1.438	0.144	1.053	0.121	1.131	0.190
Cysteine synthase	1.442	0.250	1.646	0.222	0.896	0.334
Nucleoside diphosphate kinase	1.454	0.125	1.312	0.150	0.506	0.070
Universal stress protein UspA and related nucleotide-binding proteins	1.455	0.099	0.822	0.056	0.573	0.064
Cold shock proteins	1.456	0.648	1.743	0.857	0.626	0.304
Succinyl-CoA ligase [ADP-forming] subunit alpha	1.460	0.602	1.465	0.578	1.121	0.466
Chaperone protein htpG	1.460	0.334	1.280	0.308	2.242	0.821
Dihydroorotate dehydrogenase	1.463	0.584	1.180	0.683	1.160	0.836
Malate dehydrogenase	1.464	0.797	1.204	0.320	0.875	0.282
ABC-type tungstate transport system, permease component	1.468	0.285	2.454	0.533	1.774	0.573
sulfite reductase (NADPH) flavoprotein subunit alpha	1.470	0.396	1.459	0.285	3.560	1.621
Citrate synthase	1.471	0.218	0.895	0.163	0.376	0.116
Phosphoadenosine phosphosulfate reductase	1.474	0.389	1.646	0.645	2.332	1.235
putative glutathione S-transferase YghU	1.476	0.397	1.523	0.256	0.973	0.194
long-chain-fatty-acid ligase	1.477	0.771	0.942	0.496	0.779	0.439
Lipoyl synthase	1.479	0.220	1.653	0.371	1.238	0.230
Orotate phosphoribosyltransferase	1.483	0.208	1.077	0.145	1.137	0.194
type II secretion pathway protein E	1.493	0.860	2.396	2.192	3.044	2.142
(Dimethylallyl)adenosine tRNA methylthiotransferase miaB	1.494	0.132	1.831	0.205	0.768	0.132
aspartate ammonia-lyase	1.504	0.335	1.213	0.191	2.521	0.605
2-amino-3-ketobutyrate coenzyme A ligase	1.510	0.329	1.578	0.316	3.336	0.926
LUXD Acyl transferase	1.527	0.528	1.231	0.370	1.379	0.440
hypothetical protein	1.534	0.190	1.539	0.216	2.010	0.283
Phosphoribosylformylglycinamidine cyclo-ligase	1.541	0.668	2.358	1.411	2.350	1.880

alkyl hydroperoxide reductase c22 protein	1.545	0.100	2.192	0.532	2.055	0.446
Thiazole synthase	1.552	0.158	1.592	0.207	2.085	0.337
anthranilate synthase component I	1.566	0.179	1.190	0.121	1.741	0.266
dihydrolipoamide succinyltransferase	1.568	0.380	1.295	0.395	3.315	1.249
Uridylate kinase	1.571	0.320	1.531	0.309	2.138	0.460
Phenylalanyl-tRNA synthetase alpha chain	1.572	0.369	1.377	0.321	1.856	0.410
thiamine biosynthesis protein ThiC	1.581	0.390	1.919	0.419	4.353	1.255
cAMP-regulatory protein	1.648	0.309	1.813	0.403	3.364	0.625
Transposase_31; Putative transposase, YhgA-like	1.658	0.335	1.532	0.799	0.765	0.150
3-ketoacyl-(acyl-carrier-protein) reductase	1.664	0.090	1.613	0.074	0.804	0.087
GTP-dependent nucleic acid-binding protein EngD	1.677	1.123	1.458	1.048	1.818	1.279
Elongation factor P-like protein	1.684	0.554	2.335	0.845	1.037	0.433
Glycyl-tRNA synthetase alpha subunit	1.718	0.168	1.131	0.118	1.588	0.253
Autonomous glycyl radical cofactor	1.724	0.463	1.975	0.519	1.279	0.385
10 kDa chaperonin	1.752	1.163	1.000	0.238	1.153	0.592
aminoacyl-histidine dipeptidase	1.760	0.986	1.130	0.104	1.897	0.476
Ribose-phosphate pyrophosphokinase	1.762	0.614	1.357	0.515	0.751	0.319
Sulfate adenylyltransferase subunit 2	1.777	0.150	1.378	0.152	1.544	0.333
50S ribosomal protein	1.802	0.722	0.821	0.279	2.013	0.747
fumarate hydratase, class I	1.846	0.292	2.391	0.664	2.126	0.492
L-threonine 3-dehydrogenase	1.874	0.446	1.807	0.445	2.623	0.701
Protein RecA	1.886	0.736	1.509	0.750	0.996	0.218
DNA-binding protein	2.031	1.452	1.256	0.222	4.703	2.486
ribosomal protein S20	2.082	2.074	2.522	2.509	1.498	1.363
Molybdenum cofactor biosynthesis protein	2.152	0.519	2.509	0.526	1.055	0.178
3-oxoacyl-[acyl-carrier-protein] synthase I	2.162	1.153	1.898	1.173	0.672	0.424
fructose 1,6-bisphosphatase II	2.537	3.077	4.349	6.085	6.988	10.905
azurin	2.796	2.386	0.865	0.285	0.625	0.075
heavy metal-(Cd/Co/Hg/Pb/Zn)-translocating P-type ATPase	2.949	0.143	0.889	0.012	0.997	0.094
regulatory ATPase RavA	4.023	2.230	1.229	0.452	0.897	0.230
Cell division protein ftsZ	4.288	4.935	5.264	6.442	5.162	7.002
Phasin_2; Phasin protein	4.990	3.994	4.017	3.935	2.167	1.604
